# Driving Decisions: Distinguishing Evaluations, Providers and Outcomes

**DOI:** 10.3390/geriatrics3020025

**Published:** 2018-05-11

**Authors:** Anne Dickerson, Elin Schold Davis, David B. Carr

**Affiliations:** 1 Department of Occupational Therapy, East Carolina University, Greenville, NC 27858, USA; 2 American Occupational Therapy Association, Bethesda, MD 20814, USA; escholddavis@aota.org; 3 Department of Medicine and Neurology, Washington University, St. Louis, MO 63110, USA; dcarr@wustl.edu

**Keywords:** driving, capacity, skill, driving evaluation, occupational therapy

## Abstract

Driving is a highly valued instrumental activity of daily living on which many older adults depend for access to their community. The demand to address driving is changing as older adults experience increasing longevity while facing medical conditions that often affect their fitness to drive. As one of the most complex of daily tasks, driving is a multifaceted issue that involves the older driver, family members, state licensing and health care practitioners. This commentary discusses potential options and strategies for making evidence-based fitness to drive decisions by differentiating between driving skills and driving capacities, and how these differences are manifested on the road. Typical service options are described using an algorithm format that suggests decision points with options and referrals for service based on the individual’s experiences and/or needs.

## 1. Introduction

Operating an automobile is a key instrumental activity of daily living, but it can become increasingly challenging with aging and co-existent medical disease. There are almost 40,000,000 licensed older adult drivers over the age of 65 years [1]. Unfortunately, each year over 200,000 older adults are injured in crashes [2], not including the 4000 motor vehicle deaths [3]. Crash rates per mile start to increase for drivers at age 70 and older despite the fact that many older adults restrict their driving environment to safer settings [4]. While longitudinal studies of cognitively intact older adults revealed a decline in driving performance over time [5,6], studies supporting educational interventions have shown that older adults can improve driving performance [7,8,9]. It is also important to highlight the emerging driver support anticipated through the development of automobile technologies. Some of these technologies have the potential to change how drivers interact with their vehicles, possibly providing a way of compensating for slower processing and/or physical deficits from the aging process [10]. However, while the technology enhancement is occurring rapidly, the actual translation to the motor vehicle fleet (i.e., when the technology safety features will be on the majority of vehicles) will likely take many years [11]. Since older adults primarily live in rural and suburban areas [12] and continued mobility is essential for maintaining health and quality of life [13], addressing driving and/or community mobility must become a key discussion point with primary care providers and their aging patients. This is especially true when a new diagnosis emerges with impairments that will eventually affect driving such as dementia, Parkinson’s disease, or stroke. Unfortunately, too often the messaging is not on driving safety and support but on “taking away the keys.” This negative message is inconsistent with the medical community’s role to help and leaves the patient and families left to figure out options on their own. 

There are opportunities to offer support and intervention that may reduce their crash risk and prolong older adults’ driving lifespan. This begins by starting the conversation of “transportation planning” early. The role and responsibility of physicians and primary care providers remains complex with expectations to determine whether the client should or should not continue to drive as well as determine services for intervention, transition, or cessation to a nondriver. This paper discusses providers, the data they contribute and decision points for making fitness to drive recommendations. 

## 2. Background

Health professionals interact with older adults on many levels. Some older adults attend medical clinics for wellness checks and yearly health screenings. From a medical perspective, these individuals are generally fit, are independent in activities of daily living, and typically have interest in health maintenance activities. However, other older adults have chronic conditions or illnesses that impact their physical and/or cognitive function with concomitant impairment in their higher order activities of daily living (e.g., handling money, cooking and/or medication management, independent living). Especially when those domains impact executive function, which is a key to underlying functional abilities needed for complex tasks [14], primary care providers need to consider whether there is a driving risk. 

Many medical conditions have been associated with increased crash risk [15]. Driving is an instrumental activity of daily living (IADL), along with cooking, budgeting and medication management [16]. Thus, when impairment from medical conditions affect performance so that the older adult cannot manage their own medications, finances or cooking, driving should always be discussed. In fact, driving is the most complex of IADLs and may be the first IADL that identifies the patient as having capacity deficits. However, it is often not addressed because of inadequate knowledge of what to do when it becomes a concern for the patient, caregiver, and/or clinician. 

Nevertheless, driving is a critical issue, as it is an IADL that affects both personal and **public** safety. Increased crash risk in older adults has been linked to impaired visual processing speed and attention [17]. Loss of contrast sensitivity and restrictions in visual fields due to age-associated eye diseases such as macular degeneration, cataracts and glaucoma, have been linked with impaired driving performance [18,19]. Increased crash risk has been associated with older adults that fall [20], have slow lower extremity reaction time, and reduced cervical neck range of motion [21,22,23]. Additionally, use of sedating medications typically attributed to anticholinergic side effects can reduce processing speed, impair attention with impaired driving capacity [24]. Moreover, older adults tend to take several prescription drugs, including ones that affect the central nervous system, with many of the pharmacological interactions of these drugs being unknown [24]. Thus, it is critical for the clinician to consider the patient’s impairments in relation to driving in order to refer to the appropriate services (e.g., screening, evaluation, intervention) and understand how services significantly differ depending on the patient’s capacity. Specifically, there is a distinct difference between an individual’s ability to physically operate a motor vehicle after years of practice (i.e., overlearned, automatic behavior) and the functional capacity to manage unexpected events that may happen in the dynamic driving environment (e.g., street closure, road construction, bicyclist). An updated description of Michon’s hierarchy of driving behaviors is helpful for understanding these differences as well how these differences determine which driving programs, services and providers match the older adults’ needs. Specifically, there are levels of driving behaviors defined [25]:

- Operational level: Controlling the motor vehicle through the physical actions of steering the wheel, moving or shifting gears, pressing the accelerator or brake, or using the turn signals. Draws upon skills that are overlearned and habitual so that performance of such actions is largely automatic.
- Tactical level: Executing maneuver control over the vehicle to complete a goal directed trip in response to prevailing conditions; including behaviors that are typically learned and practiced such as maintaining lane position or speed, obstacle avoidance, gap acceptance, obeying traffic signals, turning, and passing other vehicles.
- Strategic level: The general planning of a trip, including trip goals, route, and modal choice with the associated costs and risks involved; This also includes the ability to adapt plans when necessary such as changing a route due to a crash or construction, needing to make an unexpected stop (e.g., to use a bathroom), a change in a trip’s goals, or seeking help if lost.

This model can be helpful in regard to how diverse levels of impairment may impact driving safety, which visual, cognitive, and/or motor domains may be responsible, and which skills may need to be the focus for rehabilitation. 

## 3. Determining Needed Services

**Figure 1:**
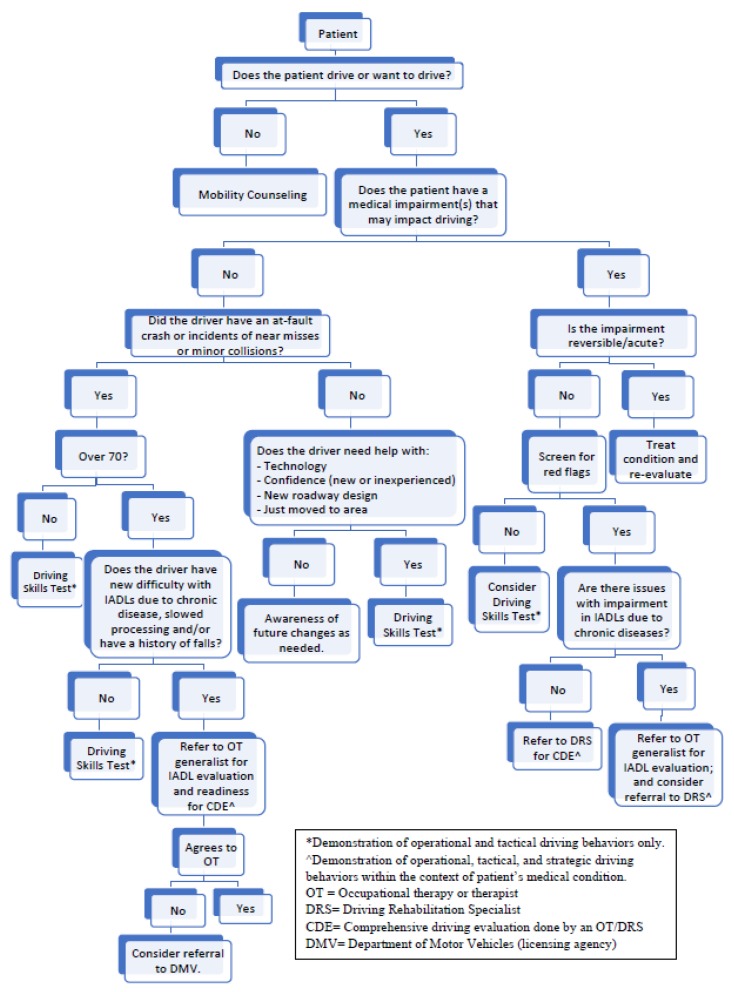
Algorithm to use to make appropriate choice of driving services for older adults when there is a question about their driving safety.

Physicians should be aware of the diversity of what constitutes a *driving evaluation*. It refers to a range of services and varies by settings, experience and background of the evaluators. Accordingly, outcomes may include lessons to correct driving errors, equipment to compensate for physical deficits, or intervention for remediation of impairment, or driving cessation. The point is, individual programs are targeted for specific audiences (e.g., driving schools teach novice drivers, state driver licensing agencies test for licensure, driving rehabilitation specialists evaluate fitness to drive), are not interchangeable and should never be compared to each other on the factor of cost. Thus, health professionals should make their best effort to refer to the services most appropriate for each individual client [26]. Figure 1 displays a suggested algorithm for clinicians to follow when making recommendations about older adults in relation to driving, highlighting what services address which level of driving behaviors (i.e., operational, tactical, strategic). 

The greatest challenge is for a clinician to determine an older adults’ medical fitness to drive. In general, physicians have not been shown to be good predictors of driving performance [27]. However, if referrals cannot be made (e.g., patient refusal, rural community, lack of specialists with driving expertise), the family clinician, physician subspecialists (e.g., neurologist, geriatrician), or a neuropsychologist may be the only source of an opinion on driving competence. While drivers themselves tend to overrate their driving capabilities, family members may be able to offer important information about noticeable problems or changes in an individual’s driving. The challenge is making a preemptive recommendation, based on diminishing capacity or impairment. When there is no prior evidence of a crash, the patient and frequently the family members may believe they still retain the ability to drive. In fact, they very likely do have the physical skills to operationally drive the vehicle and with the rule-bound environment of our roadways, be able to make the *tactical* decisions of stopping at traffic lights and remain between the lines (i.e., lane maintenance). It is the *capacity* to respond to an unexpected event in the dynamic, fast moving driving environment that needs to be evaluated for fitness to drive decisions and thus, the clinician has a moral dilemma of determining whether he or she will be over-restricting or under-restricting driving privileges. 

## 4. Diversity of Driving Evaluations and Programs

Community-based education programs are frequently found at driving schools where a licensed driving instructor (LDI) is typically certified by the state licensing agency or the Department of Education. For older adults, these programs often deliver program specific knowledge in a setting where the instructor assists the novice driver, or those interested in updating their traffic skills with the goal of identifying bad habits, modifying poor behaviors and/or seeking advice in a newly relocated area. This is based on the premise of self-awareness and the belief that these skills can be improved and maintained after a set number of sessions. It is important to understand the skill set of driving instructors, as related to hierarchy of driving behaviors, focuses on how to handle the vehicle (operational) and rules of the road (tactical) [28]. 

The LDI is an expert in driving skills and knowledge, functioning in a teaching role in how to drive a motor vehicle (i.e., operational skills) as well as the rules of the road and managing the vehicle on public highways (i.e., tactical skills). Clinicians should consider referral to these types of services for older adults that are cognitively and/or physically healthy or with slower processing due to normal aging. These services can provide reassurance that current driving skills are sufficient, find appropriate or alternative routes to avoid complex traffic situations, practice complex or new situations (e.g., unprotected left-hand turns, roundabouts), practice use of new car technologies (e.g., GPS, lane maintenance devices), or perhaps lack confidence in new environments. However, when older adults have acute medical conditions (e.g., seizures, stroke, brain injury with persistent new deficits) or chronic progressive diseases (e.g., dementia, Parkinson’s Disease) that may impair higher order IADL function, referral to either an occupational therapy generalist or an occupational therapist/Driving Rehabilitation Specialist (DRS) must be considered. A discussion of the differences between these two resources follows, since physicians may not be aware of differing skill sets and availability of occupational therapy professionals depending on practice location (e.g., rural vs. urban) or specific driving interest/experience.

Occupational therapy generalists (an occupational therapy generalist refers to the expected education of all occupational therapists. Although, they may have a non-driving related specialty such as hands, they are considered generalists as opposed to driver rehabilitation specialists) are available to physicians for (1) referral to evaluate the importance of driving as a means of community mobility to the patient; (2) evaluation of driving-related functional abilities (i.e., underlying component skills); (3) creating a “transportation plan” by planning for eventual transition through a review of community mobility needs; and (4) making recommendations regarding driving cessation/retirement. Not every medically impaired older adult requires a formal comprehensive driving evaluation and many, if not most, can be triaged appropriately by an interested and skilled occupational therapy clinician [29]. This includes obtaining information on the clients’ visual, cognitive and/or motor abilities in addition to evaluation of IADL’s. Referrals to occupational therapist/driving rehabilitation specialists must still be considered for complex or difficult cases or when mobility equipment (e.g., hand controls, wheelchair lifts) are considered.

Occupational therapists with expertise in driving rehabilitation, referred to as driver rehabilitation specialist (DRS), are often the best choice when evaluating medically complex older drivers with multi-morbidity who are not clearly cognitively compromised by their condition. Occupational therapist/DRSs’ are often based in hospital or rehabilitation settings as well as increasingly found in private practice settings. The comprehensive driving evaluation is a one-to-one specialized evaluation that typically covers both clinical assessment (e.g., physical, visual, and cognitive components) and the on-road evaluation. 

There are often concerns that the on-the-road evaluation may occur in a novel setting or in an unfamiliar car making it difficult for an older adult to pass the test. A cognitively intact older adult should be able to adapt to a different vehicle especially since a performance-based road test typically does not assess the capability to respond in an urgent situation or with critical events simply due to safety issues. In fact, passing a performance-based road test likely represents a test of overlearned skills (procedural memory), usually retained until later cognitive impairment progresses. It is one of the important distinctions in differentiating the driving evaluator (i.e., driving instructor or state licensing tester) who focuses strictly at driving skills and abilities at the operational level and tactical level. In contrast, a medically trained occupational therapist/driver rehabilitation specialist also examines abilities at the strategic level and considers driving performance within the context of additional information, such as whether the medical condition is progressive. Thus, when considering cost, a driving test that is limited to operational (automatic and overlearned skills) and tactical levels is relatively straight forwarded, determining if the individual is able to control the vehicle and follow directions (e.g., turn left at the light, park by the entrance). While the cost may be out of pocket for the patient, the comprehensive driving evaluation represents an evaluation of comprehensive skills, abilities, and capacities in light of the individual’s medical condition, not a single assessment of their ability to turn the wheel or obey the traffic light. Furthermore, this should seem like a minimal investment when the average rehabilitation recovery costs for an injury from a motor vehicle crash may be upwards of $80,000 [30].

In terms of technology, driving simulators have been validated with performance-based road tests [31] or motor vehicle crashes [32] and some programs will use simulators to evaluate fitness to drive. In fact, driving simulators have great potential for evaluation of individuals with medical conditions [33]. However, the evidence is clear that driving simulators are tools to be used by the medical professional, not in isolation of computer data output [34]. Many state drivers’ license authorities are available to evaluate medically impaired drivers. However, unless it is a mandatory reporting state, clinicians will typically use this resource as a last resort for refractor cases that refuse to be evaluated in other settings. Additionally, depending on the state, these authorities may still only be doing driving evaluation of driving skills, not evaluating driving capacity. 

The American Occupational Therapy Association (AOTA) and the Association of Driver Rehabilitation Specialists (ADED) both provide training and education to professionals that offer the comprehensive driving evaluations (CDE). These practitioners perform skilled approaches specific to driving in the context of medical impairments, with recommendations to provide only the safest mobility outcome. Evidenced-based assessments of intrinsic factors of vision, cognition and motor skills in the context of the Michon Model of driving as outlined above are typically adopted [35]. While traffic signs, rules of the road and knowledge of traffic rules, along with in traffic driving skills are usually evaluated both clinically and within the actual driving situations, it is the specialized medical knowledge of health conditions including prognosis and compensatory potential that distinguishes the LDI and a non-medical DRS from the DRS with a medical degree (OT/DRS).

Finally, clinicians have an opportunity to make recommendations to their patients to improve their quality of life and assist with productive aging. One metric of this success would be for older adults to maintain their community connectedness and autonomy by prolonging their safe and active driving. Health professionals can take an active role in suggesting educational offerings for health and medically impaired older adults. There are a myriad of educational classroom and on the road instructional courses for older adults designed to build self-awareness, offer strategies such as not driving at night and stimulate the development of an individualized transportation plan. For example, the AAA Foundation for Traffic Safety offers Roadwise Review which focuses on screening for appropriate functional skills relevant to driving [36], is available online and validated in the literature [17,19]. The Drivesharp program from Posit Science [37] pursues cognitive retraining based in part on the concept of impairments in the Useful Field of View (UFOV) that has been associated with a reduction in motor vehicle crash rates [38]. This program can be accessed through the Internet and is available on-line. UFOV training was noted in the literature to reduce dangerous driving maneuvers and improve reaction time [39,40]. ADEPT Lifelong Driver is a program that practices ever challenging high-risk crash situations with a focus on visual search strategies [41]. A recent review of these computer programs concluded that it is too early to make conclusions on their effectiveness [42]. However, they are available in a growing cadre of computer-based strategies for older adults who are interested in evaluating and/or enhancing their visual, cognitive, or motor skills related to driving. To our knowledge, classroom traffic safety courses such as those offered by AARP (Traffic Safety Course) or AAA have not been associated with a reduction in motor vehicle crash risk for older adults. Yet, some states offer insurance discounts with proof of course completion. 

On the other hand, new research has emerged that indicates education linked with on road training can improve driving skills and may allow older drivers to compensate for limitations [13,43]. In a systematic review [44], there was strong evidence that classroom type educational inventions improved awareness and self-regulation of older adults. Additionally, pairing classroom with on-road training improved driving knowledge performance, although there was no evidence for reduction in crash rate. As discussed, the occupational therapist/driving rehabilitation specialist offers an individualized one-to-one evaluation and intervention plan that may involve strategies, equipment, planning and transitioning to cessation with exploration of options to retain community mobility as a non-driver. 

In summary, there are a myriad of older driver resources, but it is important to distinguish the target audience for each resource appropriately in order to reap the benefits. This is illustrated in Spectrum of Driver Services (A Framework for comparing and understanding the range of programs and services that address driving developed under the American Occupational Therapy Association’s cooperative agreement with the National Highway Safety Administration. Supplementary), a joint document developed by AOTA and ADED [26]. Although the era of driverless cars is here, widespread limitations exist that include crash risk, insurability, and consumer confidence. It is likely that older adults will be operating motor vehicles for the near future and clinicians can play an important role in maintaining, evaluating, and/or improving driving skills.

## Supplementary Materials

The following are available online at http://www.mdpi.com/2308-3417/3/2/25/s1. Table S1: Spectrum of Driver Services: Right Services for the Right People at the Right Time, Table S2: Spectrum of Driver Rehabilitation Program Services
