# Supplementary material for: Driving Decisions: Distinguishing Evaluations, Providers and Outcomes
Source: Geriatrics (Basel). 2018 May 11;3(2):25. doi: 10.3390/geriatrics3020025 (PMC6319243; doi:10.3390/geriatrics3020025)
Supplement: Supplementary file 1 [file geriatrics-03-00025-s001.pdf]

Spectrum of Driver Services: Right Services for the Right People at the Right Time

A description consumers and health care providers can use to distinguish the type of services needed for an older adult.

The Association for Driver Rehabilitation Specialists ADED

AOTA® The American Occupational Therapy Association, Inc.

	COMMUNITY-BASED EDUCATION		MEDICALLY-BASED ASSESSMENT, EDUCATION AND REFERRAL		SPECIALIZED EVALUATION AND TRAINING
**Program Type**	**Driver Safety Programs**	**Driving School**	**Driver Screen**	**Clinical IADL Evaluation**	**Driver Rehabilitation Programs (Includes Driver Evaluation)**
**Typical Providers and Credentials**	Program specific credentials (e.g. AARP and AAA Driver Improvement Program).	Licensed Driving Instructor (LDI) certified by state licensing agency or Dept. of Education.	Health care professional (e.g., physician, social worker, neuropsychologist).	Occupational Therapy Practitioner (Generalist or Driver Rehabilitation Specialist#).<br>Other health professional degree with expertise in Instrumental Activities of Daily Living (IADL).	Driver Rehabilitation Specialist#, Certified Driver Rehabilitation Specialist*, Occupational Therapist with Specialty Certification in Driving and Community Mobility+.
**Required Provider's Knowledge**	Program specific knowledge.<br>Trained in course content and delivery.	Instructs novice or relocated drivers, excluding medical or aging conditions that might interfere with driving, for purposes of teaching / training / refreshing / updating driving skills.	Knowledge of relevant medical conditions, assessment, referral, and / or intervention processes.<br>Understand the limits and value of assessment tools, including simulation, as a measurement of fitness to drive.	Knowledge of medical conditions and the implication for community mobility including driving. Assess the cognitive, visual, perceptual, behavioral and physical limitations that may impact driving performance.<br>Knowledge of available services.<br>Understands the limits and value of assessment tools, including simulation, as a measurement of fitness to drive.	Applies knowledge of medical conditions with implications to driving.<br>Assesses the cognitive, visual, perceptual, behavioral and physical limitations that may impact driving performance.<br>Integrates the clinical findings with assessment of on-road performance.<br>Synthesizes client and caregiver needs, assist in decisions about equipment and vehicle modification options available.<br>Coordinates multidisciplinary providers and resources, including driver education, health care team, vehicle choice and modifications, community services, funding / payers, driver licensing agencies, training and education, and caregiver support.
**Typical Services Provided**	1) Classroom or computer based refresher for licensed drivers: review of rules of the road, driving techniques, driving strategies, state laws, etc.<br>2) Enhanced self-awareness, choices, and capability to self-limit.	1) Enhance driving performance.<br>2) Acquire driver permit or license.<br>3) Counsel with family members for student driver skill development.<br>4) Recommend continued training and / or undergoing licensing test.<br>5) Remedial Programs (e.g., license reinstatement course for teens / adults, license point reduction courses).	1) Counsel on risks associated with specific conditions (e.g., medications, fractures, post-surgery).<br>2) Investigate driving risk associated with changes in vision, cognition, and sensory-motor function.<br>3) Determine actions for the at-risk driver:<br>• Refer to IADL evaluation, driver rehabilitation program, and / or other services.<br>• Discuss driving cessation; provide access to counseling and education for alternative transportation options.<br>4) Follow reporting / referral structure for licensing recommendations.	1) Evaluate and interpret risks associated with changes in vision, cognition, and sensory-motor functions due to acute or chronic conditions.<br>2) Facilitate remediation of deficits to advance client readiness for driver rehabilitation services.<br>3) Develop an individualized transportation plan considering client diagnosis and risks, family, caregiver, environmental and community options and limitations:<br>• Discuss resources for vehicle adaptations (e.g., scooter lift).<br>• Facilitate client training on community transportation options (e.g., mobility managers, dementia-friendly transportation).<br>• Discuss driving cessation. For clients with poor self-awareness, collaborate with caregivers on cessation strategies.<br>• Refer to driver rehabilitation program.<br>4) Document driver safety risk and recommended intervention plan to guide further action.<br>5) Follow professional ethics on referrals to the driver licensing authority.	Programs are distinguished by complexity of evaluations, types of equipment, vehicles, and expertise of provider.<br>1) Navigate driver license compliance and basic eligibility through intake of driving and medical history.<br>2) Evaluate and interpret risks associated with changes in vision, cognition, and sensory-motor functions in the driving context by the medically trained provider.<br>3) Perform a comprehensive driving evaluation (clinical and on-road).<br>4) Advise client and caregivers about evaluation results, and provides resources, counseling, education, and / or intervention plan.<br>5) Intervention may include training with compensatory strategies, skills, and vehicle adaptations or modifications for drivers and passengers.<br>6) Advocate for clients in access to funding resources and / or reimbursement.<br>7) Provide documentation about fitness to drive to the physician and / or driver-licensing agency in compliance with regulations.<br>8) Prescribe equipment in compliance with state regulations and collaborate with Mobility Equipment Dealer^ for fitting and training.<br>9) Present resources and options for continued community mobility if recommending driving cessation or transition from driving.<br>Recommendations may include (but not restricted to): 1) drive unrestricted; 2) drive with restrictions; 3) cessation of driving pending rehabilitation or training; 4) planned re-evaluation for progressive disorders; 5) driving cessation; 6) referral to another program.
**Outcome**	Provides education and awareness.	Enhances skills for healthy drivers.	Indicates risk or need for follow-up for medically at-risk drivers.		Determines fitness to drive and provides rehabilitative services.

#DRS – Health professional degree with specialty training in driver evaluation and rehabilitation. *CDRS – Certified Driver Rehabilitation Specialist-Credentialed by ADED (Association for Driver Rehabilitation Specialists). +SCDCM – Specialty Certified in Driving and Community Mobility by AOTA (American Occupational Therapy Association).
^Quality Approved Provider by NMEDA (National Mobility Equipment Dealers Association).

Driver Rehabilitation Programs: Defining Program Models, Services, and Expertise.
Occupational Therapy In Health Care, 28(2):177–187, 2014

Spectrum of Driver Rehabilitation Program Services

A description consumers and health care providers can use to distinguish the services provided by driver rehabilitation programs which best fits a client's need.

The American Occupational The Association, Inc.

Program Type	DRIVER REHABILITATION PROGRAMS<br>Determine fitness to drive and / or provide rehabilitative services.		
Levels of Program and Typical Provider Credentials	**BASIC**<br>Provider is a Driver Rehabilitation Specialist (DRS)# with professional background in occupational therapy, other allied health field, driver education or a professional team of CDRS or SCDCM with LDI**.	**LOW TECH**<br>Driver Rehabilitation Specialist#, Certified Driver Rehabilitation Specialist*, Occupational Therapist with Specialty Certification in Driving and Community Mobility+, or in combination with LDI.<br>Certification in Driver Rehabilitation is recommended as the provider for comprehensive driving evaluation and training.	**HIGH TECH**<br>Driver Rehabilitation Specialist#, Certified Driver Rehabilitation Specialist*, Occupational Therapist with Specialty Certification in Driving and Community Mobility+.<br>Certification in Driver Rehabilitation is recommended as the provider for comprehensive driving evaluation and training with advanced skills and expertise to complete complex client and vehicle evaluation and training.
Program Service	Offers driver evaluation, training and education.<br>May include use of adaptive driving aids that do not affect operation of primary or secondary controls (e.g., seat cushions or additional mirrors).<br>May include transportation planning (transition and options), cessation planning, and recommendations for clients as passengers.	Offers comprehensive driving evaluation, training and education, with or without adaptive driving aids that affect the operation of primary or secondary controls, vehicle ingress / egress, and mobility device storage / securement. May include use of adaptive driving aids such as seat cushions or additional mirrors.<br>At the Low Tech level, adaptive equipment for primary control is typically mechanical. Secondary controls may include wireless or remote access.<br>May include transportation planning (transition and options), cessation planning, and recommendations for clients who plan to ride as passengers only.	Offers a wide variety of adaptive equipment and vehicle options for comprehensive driving evaluation, training and education, including all services available in Low Tech and Basic programs. At this level, providers have the ability to alter positioning of primary and secondary controls based on client's need or ability level.<br>High Tech adaptive equipment for primary and secondary controls includes devices that meet the following conditions:<br>1) capable of controlling vehicle functions or driving controls, and<br>2) consists of a programmable computerized system that interfaces / integrates with an electronic system in the vehicle.
Access to Driver's Position	Requires independent transfer into OEM^ driver's seat in vehicle.	Addresses transfers, seating and position into OEM^ driver's seat. May make recommendations for assistive devices to access driver's seat, improved positioning, wheelchair securement systems, and / or mechanical wheelchair loading devices.	Access to the vehicle typically requires ramp or lift and may require adaptation to OEM driver's seat. Access to driver position may be dependent on use of a transfer seat base, or clients may drive from their wheelchair. Provider evaluates and recommends vehicle structural modifications to accommodate products such as ramps, lifts, wheelchair and scooter hoists, transfer seat bases, wheelchairs suitable to utilize as a driver seat, and / or wheelchair securement systems.
Typical Vehicle Modification: Primary Controls: Gas, Brake, Steering	Uses OEM^ controls.	Primary driving control examples:<br>A) mechanical gas / brake hand control;<br>B) left foot accelerator pedal;<br>C) pedal extensions;<br>D) park brake lever or electronic park brake;<br>E) steering device (spinner knob, tri-pin, C-cuff).	Primary driving control examples (in addition to Low Tech options):<br>A) powered gas / brake systems;<br>B) power park brake integrated with a powered gas / brake system;<br>C) variable effort steering systems;<br>D) reduced diameter steering wheel, horizontal steering, steering wheel extension, joystick controls;<br>E) reduced effort brake systems.
Typical Vehicle Modification: Secondary Controls	Uses OEM^ controls.	Secondary driving control examples:<br>A) remote horn button;<br>B) turn signal modification (remote, crossover lever);<br>C) remote wiper controls;<br>D) gear selector modification;<br>E) key / ignition adaptions.	Electronic systems to access secondary and accessory controls.<br>Secondary driving control examples (in addition to Low Tech options):<br>A) remote panels, touch pads or switch arrays that interface with OEM^ electronics;<br>B) wiring extension for OEM^ electronics;<br>C) powered transmission shifter.

#DRS - Health professional degree with specialty training in driver evaluation and rehabilitation, *CDRS – Certified Driver Rehabilitation Specialist – Credentialed by ADED (Association for Driver Rehabilitation Specialists). +SCDCM – Specialty Certified in Driving and Community Mobility by AOTA (American Occupational Therapy Association) ^OEM – Original Equipment installed by Manufacturer.
**LDI-licensed driving instructor.

Driver Rehabilitation Programs: Defining Program Models, Services, and Expertise.
Occupational Therapy In Health Care, 28(2):177–187, 2014
